# The Dynamics of Trophic Cascades on Phytoplankton Induced by Mesozooplankton in Coastal Water, Daya Bay, Northern South China Sea

**DOI:** 10.3390/microorganisms12101982

**Published:** 2024-09-30

**Authors:** Bingqing Liu, Mianrun Chen, Chunsheng Wang

**Affiliations:** 1 School of Oceanography, Shanghai Jiao Tong University, Shanghai 200030, China; 2 South China Sea Institute of Planning and Environmental Research/Nansha Islands Coral Reef Ecosystem National Observation and Research Station, State Oceanic Administration, Guangzhou 510300, China; 3 Southern Marine Science and Engineering Guangdong Laboratory (Zhuhai), Zhuhai 519000, China; 4 Key Laboratory of Marine Ecosystem Dynamics, Second Institute of Oceanography, Ministry of Natural Resources, Hangzhou 310012, China

**Keywords:** mesozooplankton, Daya Bay, phytoplankton, trophic cascades, clearance rate, ciliates

## Abstract

Daya Bay, a semi-enclosed bay in the northern South China Sea and to the east of the Pearl River Estuary, is rich in biological resources and diverse habitats. Current research on mesozooplankton in Daya Bay has mainly focused on aspects such as species composition, biomass, and biodiversity in the zooplankton community. However, there is limited research on the top-down effects of mesozooplankton on prey communities. This study conducted seasonal in-situ cultivation experiments from 2015 to 2017. By combining mesozooplankton grazing experiments and microzooplankton dilution experiments, the mesozooplankton clearance rate and trophic cascading effect on low trophic levels were calculated. Results showed evident mesozooplankton selective feeding behavior and corresponding trophic cascades with seasonal variations, these being significantly higher in the spring and summer and lower in the autumn and winter. Different sizes of phytoplankton showed significant differences; large-sized phytoplankton received high feeding rates but low trophic cascades by mesozooplankton, while the opposite was true for small-sized phytoplankton. Trophic cascades contribute in three ways: offsetting direct grazing mortality, changing prey community structure via its effects on different phytoplankton sizes, and reducing ciliate grazing impacts at an average of 14.4 ± 7.8%, maintaining around 70% ciliate grazing impacts in nature. The composition of mesozooplankton was the primary reason for explaining feeding preferences, including size selectivity and omnivory. For instance, high cladoceran abundance caused high feeding rates while, on the other hand, high omnivorous copepods abundance caused high trophic cascades on small-sized phytoplankton. General additive model (GAM) analysis revealed that the changes in trophic cascades were highly dependent on temperature, ciliate abundance, mesozooplankton feeding rates on ciliates, and ciliate feeding rates on phytoplankton. The significance of this study lies in its contribution to providing valuable insights into the role of mesozooplankton in the marine food web and their impact on lower trophic levels. In addition, the findings can help inform the management and conservation of marine ecosystems, as well as guide future research in this field.

## 1. Introduction

Mesozooplankton, with body sizes ranging from 0.2 to 20 mm [1], play an essential role in marine ecosystems by transporting matter and energy from lower trophic levels to higher trophic levels, thereby connecting the classic marine food chain [2,3] and linking the microbial loop by preying on ciliates and heterotrophic dinoflagellates [4]. On average, mesozooplankton consume about 12% of marine primary production annually [5]. In contrast, microzooplankton consume an average of 67% of marine primary products daily [6], indicating that the primary group in grazing phytoplankton is not mesozooplankton, but microzooplankton [5]. On the other hand, studies [7,8] have revealed that mesozooplankton consume a significant amount of microzooplankton in the ocean [9]. Given that microzooplankton are the main consumers of phytoplankton, omnivorous and carnivorous mesozooplankton can reduce the feeding pressure on phytoplankton caused by microzooplankton. This suppression of microzooplankton by mesozooplankton feeding can cause trophic cascades, and can sometimes even lead to an increase in numbers in the phytoplankton community [10,11,12].

Trophic cascades can effectively explain the negative feeding rate of mesozooplankton on phytoplankton, especially on small particulate plankton in mesozooplankton grazing experiments [11,13]. Currently, researchers can calculate the trophic cascades induced by mesozooplankton feeding through a combination of mesozooplankton grazing experiments and microzooplankton dilution experiments [11,13]. Based on Frost’s (1972) experimental method and a calculation of the copepod feeding rate [14], Nejstgaard et al. (2001) introduced microzooplankton dilution experiments and parameters (a correction for the loss of microzooplankton feeding prey in the experimental group) to correct the errors caused by the effects of trophic cascade on the experiments [11]. Chen et al. (2013) constructed a binary regression model to interpret the relationship between the microzooplankton grazing rate and the concentration of both microzooplankton and phytoplankton; this binary regression equation can be substituted into the calculation of microzooplankton grazing rates in mesozooplankton grazing experiments [15].

To date, research on trophic cascade effects generated by plankton in oceans has been limited [13,15,16,17,18,19]. The dynamics of trophic cascades and their influential factors are still unclear. Chen and Liu (2011) have indicated that the trophic cascades induced by the calanoid copepod, *Acartia erythraea*, on the chain-forming diatom, *Skeletonema costatum*, in the presence of the heterotrophic dinoflagellate, *Protoperidinium bipes*, were mediated by the concentrations of diatoms in laboratory experiments, but the most important biological factors determining the strength of trophic cascades were the ingestion rates of both the copepods and dinoflagellates on the diatoms [20]. Chen et al. (2013) further tested in laboratory experiments if the strengths of trophic cascades were different when induced by different mesozooplankton species [15]. They found that the copepod *Parvocalanus crassirostris* in the experiments was the most carnivorous species and caused the strongest cascading effect, while the marine cladoceran, *Penilia avirostris,* generally caused a negative trophic cascade since it was incapable of capturing the intermediate grazers due to competition with microzooplankton. The strengths induced by copepods with broad feeding habits, like *Temora turbinata* and *A. erythraea,* primarily depends on the concentrations of microzooplankton and phytoplankton as the copepods can switch their preying behaviors to meet their basic energy requirements. Chen et al. (2021) showed that the strength of trophic cascades was almost equal to the grazing pressure of mesozooplankton on phytoplankton in an estuarine water [13]. This meant that they canceled each other out, resulting in the force of mesozooplankton on phytoplankton being zero in many cases, although this depended on the degree of omnivory in the mesozooplankton community, that is, the community composition of mesozooplankton.

In this study, we further tested the dynamics of trophic cascades in coastal water, with the aim of further understanding the factors that affect the trophic cascade effects. We conducted seasonal grazing experiments in a coastal station in Daya Bay. Daya Bay is located in the north of the South China Sea and east of the Pearl River Estuary. It is a semi-enclosed bay characterized by a subtropical climate [21]. Its average water depth is 10 m [21]. Daya Bay is rich in marine biological resources. There are mangrove forests and coral ecosystems, providing various habitats for marine life. The species composition of marine plankton in Daya Bay varies significantly with seasons [22]. Most studies on the zooplankton community in Daya Bay have focused on aspects such as the seasonal, inter-annual, and spatial dynamics of community composition, species abundance, and zooplankton biomass [22,23,24,25,26]. Studies on planktonic trophic interactions were relatively scarce [26]. By investigating the trophic cascade effect, we can gain a better understanding of the interactions between different trophic levels and the impact of mesozooplankton on the overall ecosystem dynamics. This knowledge is of great significance for the management and conservation of marine resources, as well as for predicting the responses of marine ecosystems to environmental changes. Additionally, this study contributes to the existing knowledge on zooplankton ecology in Daya Bay, filling a gap in our understanding of the trophic relationships in this region. The results can serve as a valuable reference for future studies and assist in the development of management strategies to maintain the health and stability of the marine ecosystem in Daya Bay and similar areas.

## 2. Materials and Methods

### 2.1. Field Samplings and Experimental Station

**Figure 1:**
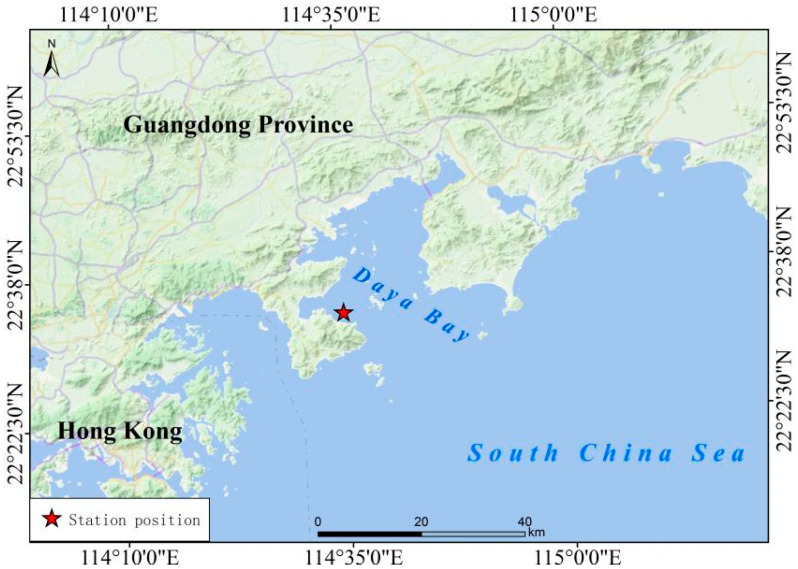
The location of the experimental station.

This study classified January to March as winter, April to June as spring, July to September as summer, and October to December as autumn, depending on regular average air temperature. There was a total of eight cruises from 2015 to 2017, which were specifically in spring 2015, summer 2015, autumn 2015, winter 2016, summer 2016, autumn 2016, winter 2017, and spring 2017. There was one experimental station with a latitude and longitude of 22°33′48.6″ N and 114°35′0.6″ E (Figure 1).

In this experimental station, we measured environmental parameters, collected field seawater for further laboratory analysis, including size-fractionated Chlorophyll *a* (Chl *a*) and planktonic biomass. We also collected field water samples and trawled mesozooplankton for the mesozooplankton grazing experiments and the microzooplankton dilution experiments in a shore-based laboratory near the station. Temperature and salinity were directly measured in-situ by a multi-parameter (YSI-SM556), with duplicate determinations of parameters to ensure accuracy. Samples for determined Chl *a* and planktonic biomass were collected by a 20 L carboy from the sub-surface (0.5 m) of the station. The water samples for analyses of Chl *a* and microzooplankton biomass and for the mesozooplankton grazing experiments and the microzooplankton dilution experiments were pre-filtered through a 200 μm sieve to remove mesozooplankton. The other part of the water was filtered through a 0.8 μm pore size bag filter, and the resulting water sample was particulate free seawater, which was used for the microzooplankton dilution experiments.

To measure size-fractionated Chl *a*, duplicated 250–500 mL seawater filtered by the sieve was taken and passed through 20-μm pore nylon filter membranes, a 2 μm pore polycarbonate membranes, and 0.7 μm pore GF/F glass fiber membranes to obtain microphytoplankton, nanophytoplankton, and picophytoplankton, respectively. The filter membranes were placed in 5 mL of 90% acetone and stored in darkness within 24 h to extract chlorophyll *a* from the filter membrane. Then the fluorescence values of the extractions were measured using a fluorescence analyzer (Triology Model 7200, Turner Designs, CA, USA). Concentrations of POC were measured to determine the Chl:C ratio of this study. Duplicated 1~3 L seawater was taken and filtered on pre-weighed GF/F glass fiber membranes, which were stored and dried before analysis. The carbon amount of the membranes was analyzed by an Elemental analyzer (EURO EA3000, EuroVector S.p.A., Milano, Italy).

To determine the concentration of microzooplankton, duplicated 100 mL seawater was taken and fixed with 5 mL of Lugol’s acid reagent and then stored for further analysis. Before the analysis, the samples were settled using a plankton settler for 12 h, and then concentrated to 5 mL. The concentrated samples were then analyzed by frequent image technology using a FlowCAM (VS-IV, Fluid Imaging Technologies, ME, USA) to determine the abundance of microzooplankton (represented by ciliates in this study) in the sample. The estimated spherical diameter (ESD) of the ciliate cell was also recorded to estimate the volume of the cell. Then an empirical equation (log pg C cell^−1^ = −0.639 + 0.984 × log V (μm^3^) for aloricate ciliates and log pg C cell^−1^ = −0.168 + 0.841 × log V (μm^3^) for loricate ciliates) was employed to transform the cell value to the carbon amount [27] (Menden-Deuer and Lessard, 2000).

To determine the composition of mesozooplankton, samples were trawled by a 200-μm mesh size plankton net with an inner diameter of 0.5 m and equipped with a flow meter in the mouth of the net. The collected mesozooplankton samples were concentrated to a volume of 500 mL. An amount of 100 mL from the concentrated sample was filtered on a pre-weighed PC membrane with a pore size of 10~20 μm. The membranes were then dried in an oven at 60 °C at the laboratory for 4 h and weighed to determine the dry-weight of mesozooplankton. The rest net tow samples were fixed with 5% formalin solution for further analysis of assemblage abundance and composition. Duplicated 10~100 mL sub-samples from the fixed samples were then analyzed for mesozooplankton species composition. Counting and species identification of mesozooplankton were performed using a stereomicroscope (SMZ-168 series, Motic, Xiamen, China) and an optical microscope (BH-I type, Olympus, Tokyo, Japan).

### 2.2. Bottle Incubation Experiments

#### 2.2.1. Mesozooplankton Grazing Experiments

Details of mesozooplankton have been described in Chen et al. (2021) [13]. Briefly, the 200-μm pre-filtered seawater was filled into six 2.4-L polycarbonate (PC) bottles, among which three bottles were used as control group and three as the treatment group. For the treatment group, a certain amount of mesozooplankton obtained from the net tow (using the same net tow method as above) was added to the bottles. To determine the added amount, duplicate same volumes by a 50-mL beaker were taken and filtered through a pre-weighed PC membrane, and the membranes were dried and weighed to determine the mesozooplankton dry weight. After 24 h of incubation, mesozooplankton from the treatment group were removed by a 200-μm filtered sieve. The 250–500 mL water samples from all bottles were filtered on different pore sizes of PC membrane to measure Chl *a* concentrations, using the same method as above. At the same time, duplicate 100 mL water samples were taken and fixed with 5% Lugol’s acid for further measuring of the abundance of ciliates, using the same method as above.

#### 2.2.2. Microzooplankton Dilution Experiments

The methods for the microzooplankton dilution experiment were developed with reference to Landry and Hassett (1982) [28] and Chen et al. (2021) [13]. Briefly, a dilution series (100, 75, 50, 25, and 16.67%) was created using various ratios of ambient and filtered water to estimate the growth rates of phytoplankton and the grazing rate of microzooplankton. Duplicate amounts of ambient water were added to 1 L polycarbonate bottles with different dilution factors. The remaining space in each bottle was filled with capsule-filtered water, which was made by pre-screening ambient water through a capsule that had a built-in filter (0.8/0.2 μm Supor Membrane, AcroPak 500, Pall Corporation, New York, NY, USA) under air pressure. Before and after the 24-h incubation period, samples of the dilution mixtures were collected for measurements of size-fractionated Chl *a*, using the same method as above to measure the chlorophyll *a*. The microzooplankton grazing rate on phytoplankton ($g_{micro}$, d^−1^) was then obtained by the regression relationship between the observed net growth rate of phytoplankton and dilution factors, through establishing a linear relationship (***k_ne_*_t_** = a − b*$g_{micro}$) in “Excel” between the net growth rate ($k_{net}$, d^−1^) as the y-value of each bottle and its corresponding dilution factor “b”(100, 75, 50, 25, and 16.67%) as the x-value, where the intercept “a” indicates the intrinsic growth rate of phytoplankton and the net growth rate $k_{net}$ can be calculated by the difference in phytoplankton concentration at the end of incubation ($P_{t}$) and the initial concentration ($P_{0}$) as:

(1) $$k_{net}=\ln\frac{P_{t}}{P_{0}}$$

And then the slope of the trend line inserted by the regression relationship is the value of $g_{micro}$**.** Based on the estimated $g_{micro}$, the clearance rate of microzooplankton on phytoplankton ($f_{micro}$, mL·ind.·^−1^·d^−1^), indicating an individual gazing rate of microzooplankton, is estimated by:

(2) fmicro=gmicroNmean

where $N_{mean}$ (ind.·L^−1^) is the mean abundance or concentration of microzooplankton, which can be estimated by the change in microzooplankton abundance (represented by ciliate abundance in this study) at the end of incubation ($N_{t}$) and the initial abundance ($N_{0}$):

(3) $$N_{mean}=(N_{t}-N_{0})/\ln\frac{N_{t}}{N_{0}}$$

### 2.3. Calculation of Mesozooplankton Feeding Rate and Trophic Cascade

In this study, the feeding rate of mesozooplankton on microzooplankton ($G_{meso}$, d^−1^) is estimated with reference to the method of Frost (1972):

(4) $$G_{meso}=K_{C}-K_{T}=\frac{\ln\left(\frac{N_{C}}{N_{0}}\right)}{t}-\frac{\ln\left(\frac{N_{T}}{N_{0}}\right)}{t}\;$$

where $K_{C}$ and $K_{T}$ (d^−1^) are the growth rate of microzooplankton in the control group and in the experimental group, respectively. $N_{0}$, $N_{C}$, and $N_{T}$ (ind.·L^−1^) are the individual abundance of microzooplankton at the beginning of the mesozooplankton incubation experiment, in the control group (no mesozooplankton feeders) and treatment group (containing mesozooplankton feeders) at incubation time t which was 1 day (d) in this study, respectively.

The clearance rate of mesozooplankton on microzooplankton ($F_{meso}$, L·mg^−1^·d^−1^) indicates the feeding rate per biomass of mesozooplankton (mg·L^−1^) and also indicates the volume of water being filtered by the specific weight of mesozooplankton, that is:

(5) $$F_{meso}=\frac{V}{dw}\times G_{meso}$$

where V is the volume (L) of the culture bottle, dw is the dry weight (mg) of mesozooplankton in the bottle. And the corresponding ingestion rate ($I_{meso}$, ind.·mg^−1^·d^−1^), indicating the microzooplankton individuals ingested by the specific weight of mesozooplankton, is:

(6) $$I_{meso}=N_{mean}\times F_{meso}$$

Since the grazing rate of mesozooplankton ($g_{meso}$, d^−1^) on phytoplankton is influenced by trophic cascades, we needed to calculate the trophic cascading rate first. The calculation method used was based on Chen et al. (2021) [13]. In the treatments of mesozooplankton grazing experiments, the direct grazing rate of mesozooplankton on phytoplankton is:

(7) $$g_{meso}=k_{C}-k_{T}+g_{micro\_C}-g_{micro\_T}=\frac{\ln\left(\frac{P_{C}}{P_{T}}\right)}{t}+TC$$

where $k_{C}$ and $k_{T}$ (d^−1^) are the net growth rate of phytoplankton in the control group and in the treatment group, respectively. $P_{C}$ and $P_{T}$ are the concentration of phytoplankton at 1-day incubation in the control group and in the treatment group, respectively. The difference of the net growth rate of phytoplankton between controls and treatment ($k_{C}-k_{T}=\frac{\ln\left(\frac{P_{C}}{P_{T}}\right)}{t}$) means a log response of phytoplankton to introduction of mesozooplankton. $g_{micro\_C}$ and $g_{micro\_T}$ indicate the grazing rate of microzooplankton on phytoplankton ($g_{micro}$, d^−1^) in the control group and the treatment group, respectively. And these rates could be estimated from the corresponding microzooplankton dilution experiment (as described above).

The difference between $g_{micro\_C}$ and $g_{micro\_T}$ is indirectly caused by mesozooplankton, that is, the strength of the trophic cascade effect, which is the trophic cascade rate ($TC=g_{micro\_C}-g_{micro\_T}$). This is manifested as the effect of mesozooplankton indirectly promoting the growth of phytoplankton through predating microzooplankton [13,15]. A weight-specific ***TC*** (d^−1^·mg^−1^) indicating the strength of trophic cascades among different experiments is estimated by dividing the *TC* by the dry weight of mesozooplankton introduced to the incubation bottles, as shown in the Result section. As the function of the mesozooplankton grazing rate (causing phytoplankton to be directly reduced) is opposite to the effect of trophic cascades (causing phytoplankton to be indirectly increased), we introduce another concept called “grazing mortality” (expressed as the negative grazing rate **−**$g_{meso}$) for phytoplankton by mesozooplankton to intuitively compare the opposite strengths between the grazing rate ($g_{meso}$) and trophic cascades (***TC***).

In the mesozooplankton feeding experiments, $g_{micro\_C}$ and $g_{micro\_T}$ cannot be directly obtained, but they can be estimated based on the microzooplankton grazing rate ($g_{micro}$) derived from the microzooplankton dilution experiments (Equations (2) and (3)) by multiplying the microzooplankton clearance rate ($f_{micro}$) and the mean concentration of microzooplankton ($N_{mean}$) in either treatments or controls of the mesozooplankton incubation bottles.

Since the grazing rate of mesozooplankton ($g_{meso}$) on phytoplankton is corrected by considering the trophic cascades [11] based on Equation (7), now we can calculate the clearance rate ($f_{meso}$, L·mg^−1^·d^−1^) and ingestion rate ($i_{meso},\mu gChl\cdot {mg}^{-1}\cdot d^{-1}$) of mesozooplankton on phytoplankton according to Equations (5) and (6), respectively, as the following equations:

(8) $$f_{meso}=\frac{V}{dw}\times g_{meso}$$

(9) $$i_{meso}=P_{mean}\times f_{meso}$$

where the mean phytoplankton concentration $P_{mean}$ can be estimated by the change of phytoplankton concentrations, similar to the way as with microzooplankton (Equation (3)):

(10) $$P_{mean}=(P_{t}-P_{0})/\ln\frac{P_{t}}{P_{0}}$$

In order to compare the results of different ingestion rates on microzooplankton ($I_{meso}$, ind.·mg^−1^·d^−1^) and on phytoplankton $(i_{meso},\mu gChl\cdot {mg}^{-1}\cdot d^{-1}$), the units of them are uniformed to prey carbon amount ingested by specific dry weight of mesozooplankton ($\mu gC\cdot {mg}^{-1}\cdot d^{-1}$) by multiplying the ingestion rate $I_{meso}$ to cell carbon value of ciliates (it was 1625~4822 pg C·cell^−1^) and by multiplying the ingestion rate $i_{meso}$ with the carbon:Chl ratio (it was 53 in this study based on relationship of Chl *a* concentration and carbon content), respectively.

### 2.4. Data Analysis

This study employed one-way ANOVA, independent t-tests or paired *t*-tests by SPSS 20 for differential analysis, with a confidence interval of 95%. For samples with non-normal distribution, the Mann Kendall test was used for difference analysis, also with a confidence interval of 95%. Significant differences were considered when *p* was less than 0.05, and highly significant differences were considered when *p* was less than 0.01. Generalized additive models (GAMs) were utilized to simulate the relationship between trophic cascade effects and environmental factors (such as temperature and salinity); biological factors (such as the Chl *a* concentration of different size phytoplankton and ciliate abundance); and mesozooplankton dry-weight and feeding rates (mesozooplankton and microzooplankton). GAM modeling was performed using the “mgcv” package built in R software version 4.0.5, using Gaussian family (normal distribution) and cubic regression spline to smooth parameters with 3 degrees of freedom.

## 3. Results

### 3.1. Environmental and Biological Factors

#### 3.1.1. Sea Surface Temperature (SST) and Salinity

**Figure 2:**
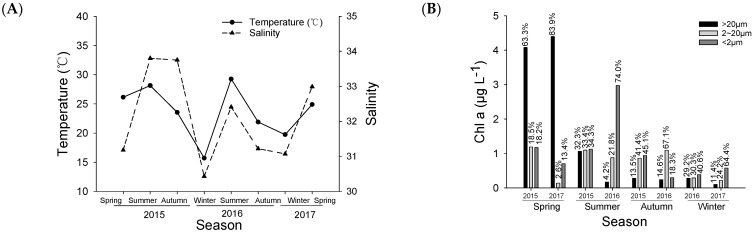

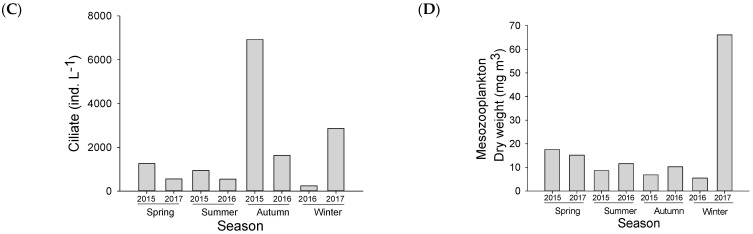
Environmental and biological factors at the experimental station from 2015 to 2017. (**A**) Temperature and salinity; (**B**) Size-fractionated Chl *a* concentrations; (**C**) Abundance of ciliates; (**D**) Mesozooplankton dry-weight.

The SST at the experimental site during the eight cruises ranged from 15.7 °C in the winter of 2016 to 29.26 °C in the summer of 2016. The average salinity was 32.11 ± 1.31, with a range from 30.43 to 33.80. Generally, the trends of SST and salinity change were basically consistent, being high in the summer and low in the winter (Figure 2A).

#### 3.1.2. Chlorophyll *a*

The Chl *a* concentration and the proportions of different sizes of phytoplankton to the total chlorophyll concentration are shown in Figure 2B. The total chlorophyll-a (Chl *a*) concentration in spring, summer, autumn, and winter was 5.84 μg·L^−1^, 3.65 μg·L^−1^, 1.86 μg·L^−1^, and 0.93 μg·L^−1^, respectively. The concentrations were significant different among all seasons (one-way ANOVA, *p* < 0.01), following a general pattern of spring > summer > autumn > winter.

In terms of the contribution of different size fractions to total Chl *a* concentration, microphytoplankton (>20 μm), nanophytoplankton (2~20 μm), and picophytoplankton (<2 μm) accounted for an average proportion of 31.55%, 29.91%, and 38.54%, respectively. Overall, there was no significant difference among the three size classes (Mann Kendall test, *p* > 0.05), suggesting that the prey conditions were dynamic and not consistent throughout the sampling period of this research. Different size classes dominated at different periods. For example, microphytoplankton was higher in the spring (63.30%~83.93%), while their proportion decreased in summer, autumn, and winter. Nanophytoplankton accounted for a high proportion of 67.11% in the autumn of 2016. On the other hand, picophytoplankton accounted for a high proportion in the summer of 2016 and winter of 2017, with 74.00% and 64.43%, respectively.

#### 3.1.3. Microzooplankton

Microzooplankton in this study is represented by ciliates. The abundance of ciliates at the experimental site is shown in Figure 2C. The average abundance of ciliates from the eight cruises was 1868 ± 275 ind.·L^−1^, ranging 240 to 6920 ind.·L^−1^. The highest value was found in the autumn of 2015, while the lowest was found in the winter of 2016.

#### 3.1.4. Mesozooplankton

The dynamics of mesozooplankton biomass, represented by dry weight data, are shown in Figure 2D. The average dry weight of mesozooplankton from the eight cruises was 17.71 ± 19.98 mg·m^−3^, ranging 5.47 (winter of 2016) to 66.14 mg·m^−3^ (winter of 2017). Generally, the dry weight was relatively higher in the spring than in the summer and autumn (one-way ANOVA, *p* < 0.05). Winter data of the two sampling years (2016 and 2017) were strikingly different, likely due to the community composition.

**Figure 3:**
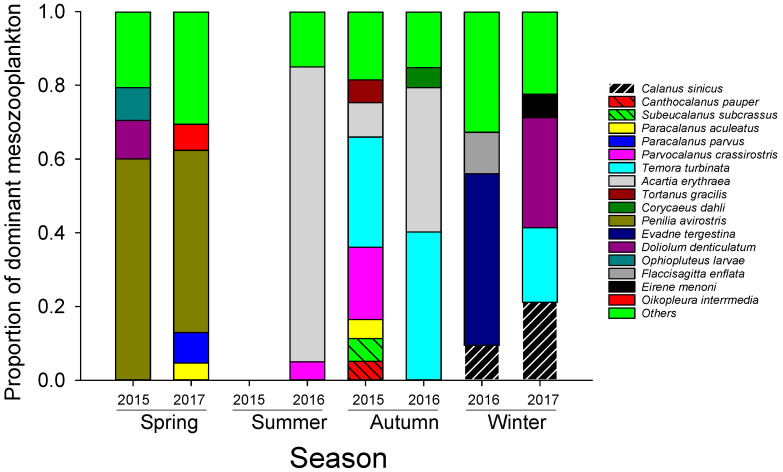
Dominant species of mesozooplankton and their proportions.

The composition of mesozooplankton species from the seventh cruise (data from summer 2015 missed) was shown in Figure 3. The composition of dominant species of mesozooplankton varied greatly in different seasons and years. The cladoceran species, *Penilia avirostris*, was primarily dominating in the spring of both different sampling years (2015 and 2017). During the summer (2016) and the autumn (2015 and 2016), the community was primarily dominated by copepod species, such as *Acartia erythaea* (highest at the summer of 2016), *Temora turbinata* (highest at the autumn of both 2015 and 2016), and *Parvocalanus crassirostris* (the second highest at the autumn of 2015 and contributed a great portion in the summer of 2016). Another copepod species, *Calanus sinicus*, was dominant in the winter of both sampling years (2016 and 2017). But the most dominant species in the winter were primarily *Evadne tergestina* (cladocerans) and *Doliolum denticulatum* (tunicates) in 2016 and 2017, respectively. Generally, the proportion of mesozooplankton in copepods was high, followed by cladocerans and tunicates *(Doliolum denticulatum* and *Oikopleura interrmedia*). Other abundant species were represented by *Paracalanus* spp. (spring 2015 and autumn 2017); *Subeucalanus subcrassus, Canthocalanus pauper*, and *Tortanus gracilis* (autumn 2017); and *Flaccisagitta enflata* (winter 2016), etc.

### 3.2. Feeding Rates

#### 3.2.1. Rates of Microzooplankton on Phytoplankton

Microzooplankton feeding rates, including the grazing rate ($g_{micro}$) of the microzooplankton community on phytoplankton, and then clearance rate (***f_micro_***) of individual ciliates on phytoplankton, derived from the microzooplankton dilution experiments are shown in Table 1. Ciliate grazing rates and clearance rates on total phytoplankton ranged 0.22~2.06 d^−1^ and ranged 0.12~2.57 mL·ind.·^−1^·d^−1^, respectively. The rates were relatively high in the spring (Paired *t*-test, *p* < 0.01) and the summer, while they were lower in the autumn and winter. No significant difference was found for the rates among different size fractions of phytoplankton (*p* > 0.05). However, we found that higher rates on microphytoplankton (>20 μm) than the other two size fractions primarily appeared in the spring, and generally the rates on the small size factions were higher than on the microphytoplankton in other seasons. Higher rates on nanophytoplankton (2–20 μm) compared to other size fractions mainly appeared in the summer and winter. In the autumn, the rates were generally higher for picophytoplankton (<2 μm).

#### 3.2.2. Mesozooplankton Clearance Rate

**Figure 4:**
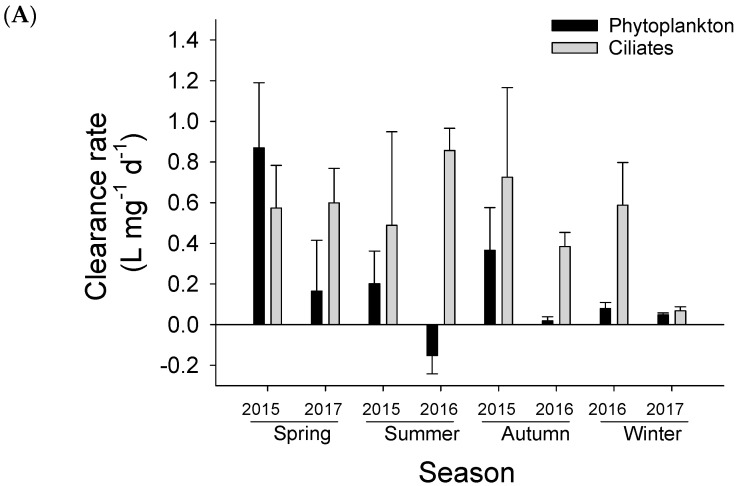

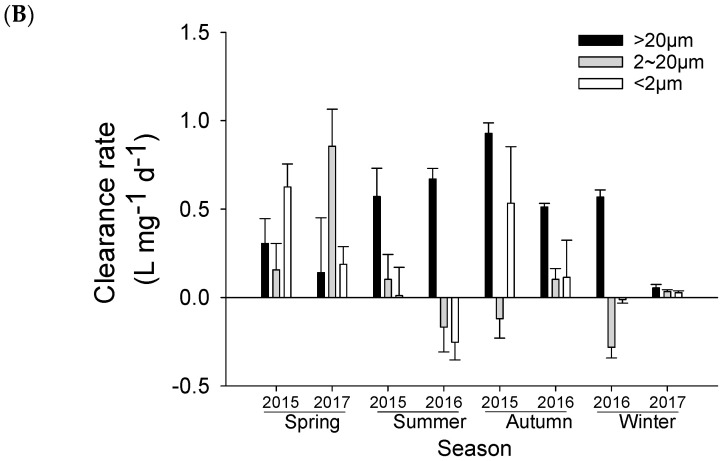
The clearance rate of mesozooplankton on (**A**) microzooplankton and total phytoplankton and on (**B**) different sizes of phytoplankton at the experimental site in four seasons from 2015 to 2017.

The clearance rates of mesozooplankton on ciliates (***F_meso_***) and total phytoplankton (***f_meso_***) at the experimental sites from 2015 to 2017 are shown in Figure 4A. In all four seasons, the average clearance rates of mesozooplankton on ciliates (***F_meso_***) ranged from 0.07 ± 0.02 to 0.86 ± 0.11 L·mg^−1^·d^−1^, with the lowest rate occurring in the winter of 2017 and the highest rate appearing in the summer of 2016. However, seasonal variation was not significant (one-way ANOVA, *p* > 0.05). Overall, the rates on ciliates were significantly higher than on phytoplankton (Paired *t*-test, *p* < 0.05), indicating an apparent feeding preference of mesozooplankton on ciliates to phytoplankton.

The average clearance rates (***f_meso_***) on phytoplankton (corrected by the effect of trophic cascades as described above) ranged from −0.15 ± 0.09 to 0.87 ± 0.32 L·mg^−1^·d^−1^, with lowest rate in the summer of 2016 and the highest rate in the spring of 2015. Overall, the average clearance rates on phytoplankton were significant higher in the spring and lower in the winter (one-way ANOVA, *F* = 14.213, *p* < 0.01).

Regarding the different size fractions of phytoplankton, the average mesozooplankton clearance rates on microphytoplankton, nanophytoplankton, and picophytoplankton ranged from 0.05 ± 0.02 to 0.93 ± 0.06 L·mg^−1^d^−1^, −0.28 ± 0.06 to 0.85 ± 0.21 L·mg^−1^d^−1^, and −0.25 ± 0.10 to 0.62 ± 0.13 L·mg^−1^d^−1^, respectively. Among them, the clearance rates of mesozooplankton were generally higher on microphytoplankton (>20 μm) than the other two size fractions (one-way ANOVA, *F* = 3.479, *p* < 0.05), except during the spring. For seasonality, rates on microphytoplankton were generally higher during the summer and the autumn, while on the other hand the rates on nanophytoplankton and picophytoplankton were generally higher during the spring. Occasionally, negative clearance rates on the two small size fractions were detected (autumn of 2015, summer, and winter of 2016), indicating that the appearance of mesozooplankton caused an increase in phytoplankton growth but it was not due to the trophic cascades as the effect of trophic cascades on the direct grazing of mesozooplankton had been corrected.

#### 3.2.3. Mesozooplankton Ingestion Rate

**Figure 5:**
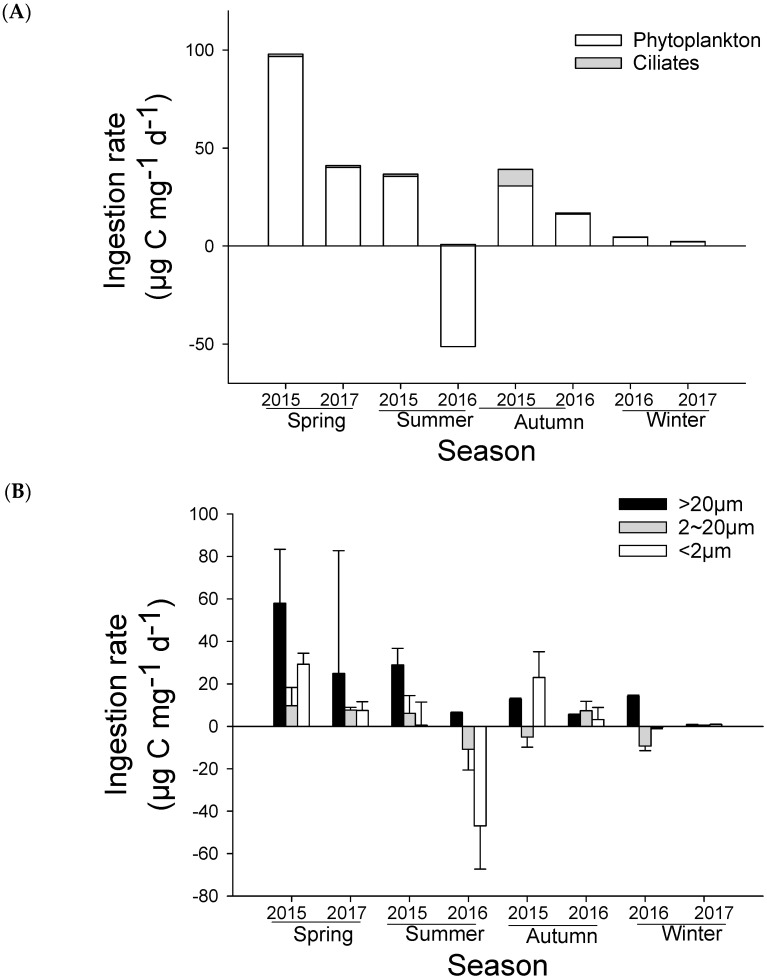
The ingestion rate of mesozooplankton on (**A**) microzooplankton and total phytoplankton and on (**B**) different sizes fractions of phytoplankton at the experimental site from 2015 to 2017.

The ingestion rate (***i_meso_***) of mesozooplankton on total phytoplankton is shown in Figure 5A. The average rates on phytoplankton ranged from −51.3 ± 26.7 to 96.8 ± 36.5 μg C·mg^−1^·d^−1^. As with the clearance rates (***f_meso_***), mesozooplankton had the highest ingestion rate in the spring of 2015 and low ingestion rates in the winter. And a negative ingestion rate was observed in the summer of 2016 due to the negative clearance rate. Compared to the rates of phytoplankton, the rates on ciliates (***I_meso_***) were particularly low due to their low concentrations in carbon unit, suggesting that phytoplankton were the main diet for mesozooplankton assemblage in terms of carbon requirement.

The ingestion rates of mesozooplankton on phytoplankton with different size fractions are shown in Figure 5B. The ranges of ingestion rate of mesozooplankton on microphytoplankton, nanophytoplankton, and picophytoplankton were 0.8 ± 0.2~57.9 ± 25.5 μg C·mg^−1^·d^−1^, −10.9 ± 9.7~9.7 ± 8.6 μg C·mg^−1^·d^−1^, and −46.9 ± 20.4~29.1 ± 5.2 μg C·mg^−1^·d^−1^, respectively. Rates on microphytoplankton were higher in the spring than other seasons and were overall higher than other size fractions in most seasons. The negative ingestion rates on total phytoplankton in the summer of 2016 were contributed to by the rates of nanophytoplankton and picophytoplankton. If no negative ingestion rates were considered, the positive rates of mesozooplankton on all size factions of phytoplankton were much higher than those on ciliates.

### 3.3. Trophic Cascades Induced by Mesozooplankton

#### 3.3.1. Trophic Cascade

**Figure 6:**
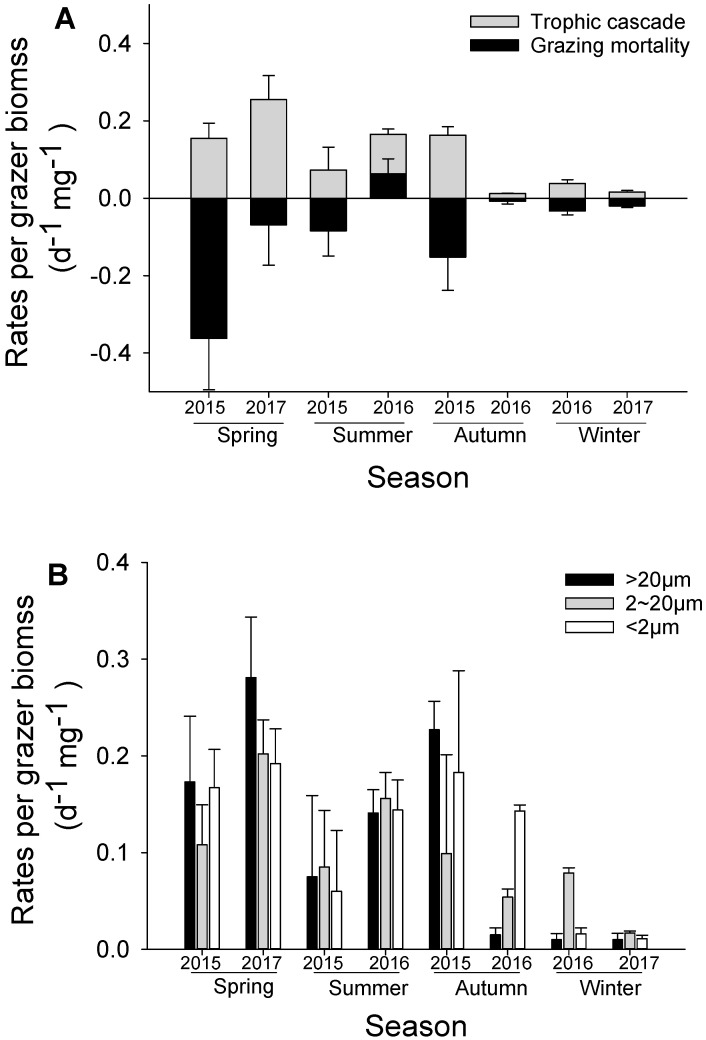

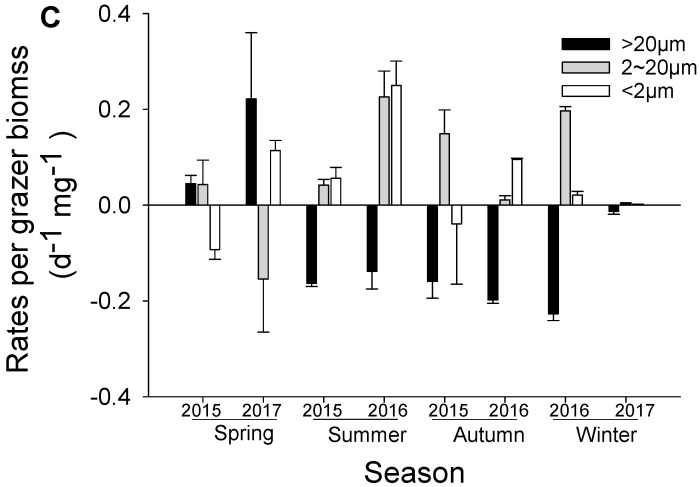
Mesozooplankton trophic cascades (**A**) on total phytoplankton and comparison to grazing mortality (expressed by negative grazing rates) by mesozooplankton on total phytoplankton (**B**) on different size fractions of phytoplankton; and (**C**) the discrepancy between the trophic cascades and the grazing mortality for different size fractions of phytoplankton at the experimental site from 2015 to 2017.

The comparison of indirect promotion (expressed as the trophic cascading rate) and direct consumption (grazing mortality) are shown in Figure 6. The range of average trophic cascading rate (***TC***) of mesozooplankton on total phytoplankton was 0.012 ± 0.001~0.255 ± 0.062 d^−1^·mg^−1^. Overall, ***TC*** exhibited a significant seasonal variation pattern (one-way ANOA, F = 7.943, *p* = 0.001), with the rates in the spring being much higher than those in other seasons and the rates in the summer being higher than those in the winter (Figure 6A). The seasonal pattern of trophic cascades was similar to that of grazing mortality (−$g_{meso}$) on phytoplankton by mesozooplankton, except in the summer of 2016 when mesozooplankton did not cause a negative grazing mortality of phytoplankton. Rates were both high in the spring and summer, whereas the rates were equally low in the winter. The comparisons of the trophic cascades and grazing mortality indicated that the effects of trophic cascades and those of grazing mortality were canceling each other out in most cases, like in the autumn and the winter. However, the situations in the spring and the summer were different between the two sampling years, as there was a rather high direct grazing mortality in the spring of 2015 and a positive grazing mortality (with the appearance of mesozooplankton causing an increase in phytoplankton) in the summer of 2016.

Although no significant difference of trophic cascades among the three size fractions was found (Figure 6B; one-way ANOVA, and *p* > 0.05), the discrepancy between the trophic cascades and the grazing mortality was significantly different among the three size fractions of phytoplankton (Figure 6C; one-way ANOVA, F = 8.842, and *p* < 0.001). There was a positive effect of mesozooplankton on microphytoplankton during the spring indicated by a positive discrepancy (trophic cascade > grazing mortality), where there were negative effects (trophic cascade < grazing mortality) during the other three seasons. By contrast, there were generally positive effects (trophic cascade > grazing mortality) of mesozooplankton on nanophytoplankton and picophytoplankton in most cases except the spring.

#### 3.3.2. GAM Analysis of Controlling Factors for Trophic Cascades

To better understand the pattern of the variations of trophic cascades, we used the Generalized Additive Model (GAM) to analyze the effects of various factors to the changes of trophic cascades, including the key environmental factors (such as temperature and salinity) and biological factors like mesozooplankton biomass, Chl *a* concentrations, ciliate abundance, mesozooplankton clearance rate (***F*_meso_**), and ingestion rate (***I_meso_***) on ciliates, and ciliate clearance rate on phytoplankton (***f_micro_***). All available factors were firstly tested together, and then those statistically non-significant factors (such as other environmental factors like pH and DO and biological factors like the percentage of different size fractions of phytoplankton and mesozooplankton feeding rates on phytoplankton) were removed from the models. Season as an important driving factor was also included in the model as a linear factor without any smooth.

**Figure 7:**
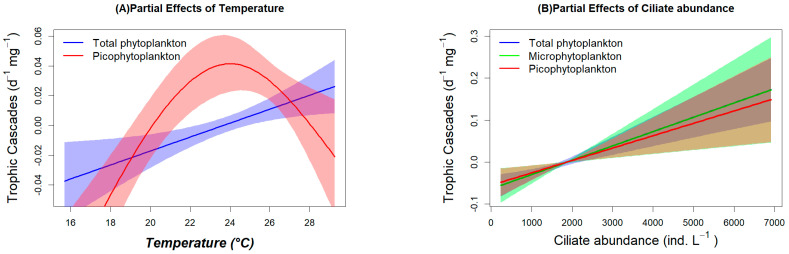

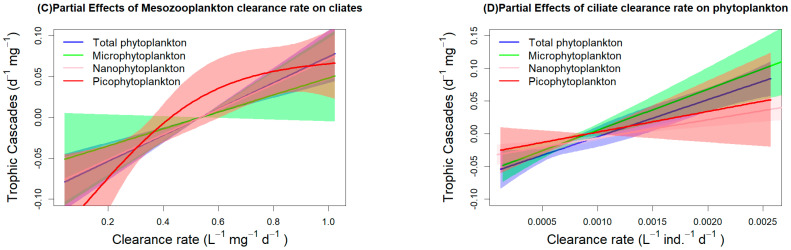
Regression relationship of partial effects (95% confidence intervals) of typical significant factors and the predicted change of trophic cascades (for Total or different size fractions of phytoplankton) via GAM analysis.

Statistically significant factors with corresponding F-test values (t-test values for the parameter of season) and significance levels and the percentage of deviation explained and the R-square of the model are shown in Table 2. The regression relationships between the change of trophic cascades and typically significant factors are shown in Figure 7. Overall, the speed of all the models used in this study were rather good, as suggested by the high values of deviance explained (%) and R^2^. Spring was a factor that significantly increased the change of trophic cascades on total phytoplankton and microphytoplankton (Chl *a* > 20 μm), whereas summer and winter were factors that led to a decline in change for nanophytoplankton (Chl *a* 2–20 μm). Temperature played a highly important role in increasing the change of trophic cascades for the total phytoplankton and exerted a non-linear influence on that for picophytoplankton (Figure 7A).

The partial effect of temperature on the change of trophic cascades for picophytoplankton was maximal when the temperature was approximately 24 °C, and this mainly occurred in spring. Salinity, as well as mesozooplankton biomass and Chl *a* concentration in environment, were significant factors influencing the change of trophic cascades, and were mainly effective for nanophytoplankton. Ciliate abundance and the ciliate clearance rate (***f_micro_***) on phytoplankton (data of different size fractions for each model) played a particularly important role, as suggested by the high F-value, significance level, and the apparently positive regression relationship (Figure 7B,D). The mesozooplankton clearance rate (***F_meso_***) and ingestion rate (***I_meso_***) on ciliates were other extremely important and conceivable factors influencing the change of trophic cascades as they directly influenced the abundance of ciliates, and high rates of them represented the omnivorous behavior of the mesozooplankton assemblage.

#### 3.3.3. Results of Trophic Cascades on Ciliate Grazing Impacts

**Figure 8:**
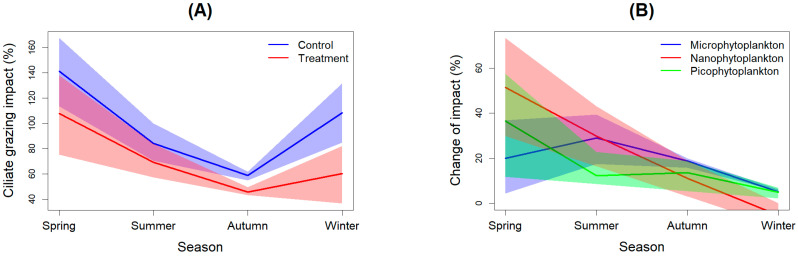
Seasonal Variation (smooth) of Ciliate Grazing Impact (with 95% Confidence): (**A**) Between Treatment and Controls; (**B**) Change per Mesozooplankton Biomass on Different Size Fractions of Phytoplankton.

Due to the influence of trophic cascades, ciliate grazing impacts (ciliate ingestion rate (***i*_micro_** = **f_micro_** × **P_mean_**) × mean ciliate abundance (**N_mean_**)/phytoplankton concentration(**P_0_**) × 100%) were significantly reduced, as evidenced by the reduced grazing impacts in treatment bottles (Figure 8A). The mean ciliate grazing impacts in controls and treatments were 98 ± 52.1% and 70.7 ± 40.3%, respectively. A distinct seasonal variation pattern emerged, with high impacts witnessed in spring and low impacts in autumn. By subtracting the impacts between controls and treatments, we discovered that mesozooplankton trophic cascades led to an average 14.4 ± 7.8% of change in terms of grazing impacts. No statistically significant difference in change was found among the three different size fractions of phytoplankton (paired-*t*-test, *p* > 0.05). However, it can be seen that trophic cascades generally caused a higher reduction of change on smaller sizes of phytoplankton than on large sizes in spring, while the opposite was true in other seasons (Figure 8B). Overall, seasonal variations were obvious, in which the average reduction level was particularly high in the spring and low in the winter (Figure 8B). Moreover, the reduction level consistently declined from spring to winter (Figure 8B).

## 4. Discussion

### 4.1. Seasonal Variations of Mesozooplankton Feeding Rates

Mesozooplankton clearance rate and trophic cascades on phytoplankton represent two diametrically opposite roles of the mesozooplankton assemblage in regulating the prey community. Such contrary functions have a profound impact on the structure and balance of the entire ecological system. The variations of these two roles are contingent upon numerous factors, including the species composition of mesozooplankton, the composition of food, and the influence of environmental elements. As a result, they demonstrate certain seasonality. For example, a high clearance rate is typically witnessed when herbivorous species predominate or when the concentration of phytoplankton is extremely low and omnivorous species need to consume a sufficient quantity of phytoplankton. Additionally, when the temperature is relatively high and the metabolism of the entire community is elevated, they adopt a low-energy-consumption method of filter feeding. On the contrary, when the trophic cascades are relatively high, carnivorous zooplankton usually hold a certain advantage at this time. Moreover, the proportion of microzooplankton in the food is relatively large, and the temperature is moderately suitable, causing omnivorous grazers to potentially seek additional nutrients by preying on microzooplankton for reproduction.

In this study, we followed the methods of Nejstgaard et al. (2001) [11] and Chen et al. (2021) [13] to correct the calculation of clearance rate of mesozooplankton in the bottle incubation method by removing the trophic cascades from the net log response of phytoplankton. Overall, the clearance rate of mesozooplankton on total phytoplankton was relatively low, with a negative value observed at one point in the summer of 2016 and relatively high values observed in the spring and autumn of 2015 (Figure 4). The negative clearance rate obtained in this study was not due to the influence of feeding on ciliates as the correction of mesozooplankton clearance rate had been made.

By comparing the clearance rates on ciliates and on phytoplankton, a feeding preference of mesozooplankton upon ciliates to phytoplankton was clearly evidenced, except for in the spring of 2015 (Figure 4A), suggesting that the assemblage of mesozooplankton was overall omnivorous in terms of its feeding proactivity. However, when comparing ingestion rates, the results showed that mesozooplankton ingestion rates were primarily contributed to by phytoplankton, particularly by microphytoplankton, while ciliates contributed a rather small portion (ranging 1.2~19.3% and a mean of 6.8%) to the total positive ingestion rate (Figure 5A). This suggests that the assemblage of mesozooplankton had a high degree of herbivory in terms of its feeding passivity due to the high carbon concentrations of the phytoplankton and low carbon concentrations of the ciliates in the water environment. Such a conclusion has certain rationality as phytoplankton play an important role in bottom-up controls of the food web. However, the contribution of animal food to the total ingestion rate could be even greater than the values we obtained. This is mainly because we lack a more detailed study of the contribution of other animal prey, such as heterotrophic dinoflagellates and small zooplankton, including copepod nauplii or smaller-sized copepods, to the total zooplankton ingestion rate. The ingestion of these prey can also lead to a certain degree of trophic cascades, which might be the reason why, despite corrections, the direct clearance rate of mesozooplankton we obtained was still negative in some cases. For example, Chen and Liu (2011) demonstrated that the copepods *Acartia* can cause a high trophic cascade on chain-forming diatom *Skeletonema* through the ingestion of the heterotrophic dinoflagellates *Protoperidinium* [20]. Therefore, we may underestimate the strength of trophic cascade in some special case when we detect the negative clearance rate.

Of course, there may be other reasons for the negative situation, such as nutrient regeneration by mesozooplankton through excretion or defecation, which have a certain promoting effect on the growth of small phytoplankton [29]. Moreover, a strong size-elective feeding behavior together with a feeding avoidance of mesozooplankton may also lead to a negative clearance rate for some phytoplankton groups. For example, a negative clearance rate on phytoplankton which contributed by the promotion of the two small size fractions of phytoplankton (nano- and picophytoplankton) was observed during the summer of 2016 (Figure 4A,B) when the concentration and portion of microphytoplankton was extremely low at this sampling time (Figure 2B). In this case, mesozooplankton proactively seek to feed on large-sized particles and avoid feeding on the two small-sized phytoplankton, meaning the latter wins the competition for nutrients, which makes them dominant in the community. Similarly, Chen et al. (2021) showed that when phytoplankton is in bloom, a negative clearance rate will be observed due to a time-lag of copepod development and a feeding avoidance of copepods to the bloom organisms [13].

In line with other studies in adjacent waters [13,30], the size-selective feeding behavior of mesozooplankton in this study was also clear, evidenced by the comparison of clearance rates towards the three size fractions of phytoplankton (Figure 4B). Generally, microphytoplankton contributed the greatest proportion (mean ± SE: 63.9 ± 22.6%) to mesozooplankton ingestion rate on total phytoplankton, followed by picophytoplankton (mean ± SE: 21.8 ± 7.7%), and then nanophytoplankton (14.3 ± 5.0%). This composition ratio was highly inconsistent with the composition ratio of food in the environment, suggesting a feeding preference for microphytoplankton (31.55% in environment) and a feeding avoidance of the two small-sized phytoplankton (29.9% for nanophytoplankton and 38.54% for picophytoplankton in environment).Researchers reported that mesozooplankton at the same station of this study and adjacent waters had a feeding preference on dinoflagellates and cryptophytes with relatively low concentration in environment but feeding avoidance to diatoms with high concentration in environment based on HPLC pigment analysis [26,31].

The feeding rates among different prey displayed different seasonal patterns and even varied in different years of the same season. Various factors will affect the mesozooplankton clearance rate, such as temperature, food concentration, and grazer composition. For example, when seawater temperature decreased, the mesozooplankton reduced their feeding rate, which resulted in low clearance rates on most prey in the winter of this study. For microphytoplankton, high clearance rates were primarily witnessed during the summer and autumn and a case in the winter of 2016, which coincided with the relatively low concentration of microphytoplankton during these periods. Meanwhile, low clearance rates occurred in two scenarios. One was in the spring when the concentration of microphytoplankton was high, and mesozooplankton did not need a very high clearance rate to obtain a relatively high feeding intake, as indicated by high ingestion rate during the spring. The other scenario was in the winter of 2017, when, despite the low concentration of phytoplankton, the entire mesozooplankton assemblage had a low feeding rate, which was primarily due to the low temperature. For nanophytoplankton and picophytoplankton, their significant contributions to mesozooplankton ingestion rate and relatively high clearance rate were primarily witnessed in the spring and the autumn, while low and negative rates primarily occurred in the summer and the winter. For ciliates, no clear seasonal pattern was found, but a particularly low clearance rate was found in the winter of 2017 (Figure 4A) and a high contribution was found in the autumn of 2015 (Figure 5A).

Mesozooplankton composition in different seasons greatly accounted for the seasonal and annual variation patterns of mesozooplankton feeding rates in this study. The composition of mesozooplankton varied greatly, and the feeding preferences of different species for phytoplankton of different sizes are different. In the spring, mesozooplankton assemblage was dominated by the caladoceran species, *Penilia avirostris* (Figure 3). This species is reported to prefer feeding on small particles because of their fine suspension-feeding behaviors [15,32,33,34]. Thus, relatively high rates on nanophytoplankton and picophytoplankton probably resulted from the feeding of this cladoceran species. Comparing the same season of the two sampling years, 2015 and 2017, we found that the clearance rates for the two small-sized phytoplankton were slightly different, which is probably due to higher proportion of *P. avirostris* and the contribution of a tunicate species, *Doliolum denticulatum,* in the year 2015, causing a higher clearance rate on picophytoplankton and higher proportion of copepod species *Paracalanus*. In the year 2017, another tunicate species, *Oikopleura interrmedia,* caused a higher clearance rate on nanophytoplankton.

In the summer of 2016 (sample of 2015 missed), mesozooplankton were predominated by the copepod species, *Acartia erythraea,* which was probably the reason for the relatively high clearance rate on ciliates and microphytoplankton, as the genera *Acartia* are mainly omnivorous with a preference for microzooplankton and large-sized phytoplankton [15,35,36]. Due to its high predation behavior, the underestimation of trophic cascades by *Acartia erythraea* through predating on heterotrophic dinoflagellates or other copepod larvae may contribute to the negative clearance rate of nanophytoplankton and picophytoplankton during this particular season.

In the autumn, the composition of mesozooplankton was slightly different between the two years. The similarity was that the assemblage was dominated by the two copepod species, *Temora turbinata* and *Acartia erythraea,* during both years. The difference was that in 2015, multiple species were jointly dominant, while in 2016, these two species were mainly in absolute dominance. The highly omnivorous species of *T. turbinata* has a broad feeding size-spectrum for particles [15]. They prefer small and immobile or slow-moving planktonic organisms and ciliates [37,38,39]. As a result, both phytoplankton (mainly nanophytoplankton and microphytoplankton) and ciliates were fed on at a high rate in this study. Numerous copepod species, such as *Parvocalanus crassirostris*, *Canthocalanus pauper*, *Subeucalanus subcrassus*, *Paracalanus aculeatus* and *Tortanus gracilis* jointly dominated the assemblage *and* complicated the feeding behavior of the assemblage in the autumn of 2015. Among these dominant species, most of which have a very high degree of carnivory and can consume a lot of animal food other than ciliates [5,15,30,40,41]. As a result, the trophic cascades induced by them might potentially be underestimated, which might also be one of the causes for the occurrence of a negative clearance rate of nanophytoplankton in this season.

The biological conditions in the two winters in this study were considerably different. Abundance of ciliates, the structure of size-fractionated Chl *a*, as well as both the biomass and community structure of mesozooplankton were markedly different between the two winters (Figure 2C,D and Figure 3). In the winter of 2016, the biomass of mesozooplankton and ciliate abundance were rather low, and the phytoplankton structures of the three different size fractions were relatively uniform. Conversely, in the winter of 2017, the biomass was rather high, and small-sized phytoplankton particles accounted for an absolute majority. In terms of the community structure of mesozooplankton, the main dominant species in the winter of 2016 were a cladoceran species, *Evadne tergestina*, arrowworms, and a small proportion of a copepod species, *Calanus sinicus*. In the winter of 2017, the main species were a tunicate species, *Doliolum denticulatum,* a copepod species, *C. sinicus*, and *T. turbinata*. These different conditions led to a slightly different clearance rate of mesozooplankton (***f_meso_***). Specifically, there was an extremely low clearance rate in 2017, while in 2016, mesozooplankton had a relatively high clearance rate for microphytoplankton, but negative rates for the two small-sized phytoplankton (as explained above). Mesozooplankton composition was likely the primary reason for explaining these results. The feeding behavior of the cladoceran species, *E. tergestina*, which prefers large-sized phytoplankton, is different to that of *P. avirostris,* which dominated in both springs. However, these different conditions resulted a similar strength of trophic cascades and grazing mortality, implying that low temperature was probably the main factor controlling the trophic effect of the mesozooplankton community during winter.

### 4.2. Variation of Trophic Cascades and Its Controlling Factors

As calculated by this study, the strength of trophic cascades was relatively high (Figure 6). High trophic cascades were recorded in the spring and summer, and the autumn of 2015, consistent to high ingestion rates on ciliates during the same period (Figure 5A). Such a seasonal pattern is similar to the adjacent water Pearl River Estuary [13]. The highest value appeared in the spring of 2017. We found that the food environment in the spring was characterized by high phytoplankton concentrations, particularly by high microphytoplankton but low ciliate abundance (Figure 2B), and omnivorous copepod species *Corycaeus dahli* and the two *Paracalanus* species played an important role for the assemblage. The clearance rate for ciliates were increasing from winter to spring but the rate for microphytoplankton was relatively low. In this scenario, mesozooplankton tended to avoid grazing phytoplankton and proactively sought predating ciliates to meet their requirement for nutrition. Chen and Liu (2011) and Chen et al. (2013) demonstrated in a laboratory that the strength of trophic cascades will be increased with increasing phytoplankton concentration due to a switch of grazer feeding behavior, because the feeding efficiency of consumers is a functional response to the change in prey densities (low clearance rate at high concentration and high clearance rate at low concentration) [15,20]. Thus, due to the intense predation by mesozooplankton in the spring in this study, the growth of microzooplankton was greatly inhibited, thus triggering a high trophic cascade effect on phytoplankton [13,18].

On the other hand, during the summer, concentration of size-suitable ingested phytoplankton (>2 μm) markedly reduced from the spring, while the species markedly contributed a great proportion in mesozooplankton assemblage. As a result, high trophic cascades together with a high clearance rate for ciliates were evidenced. There was a special case in the autumn of 2015 when ciliate abundance reached a notable maximum, while numerous omnivorous copepod species were jointly dominant in the assemblage, causing a high clearance rate on ciliate and a high trophic cascade. During the autumn of 2016, phytoplankton were primarily dominated by nanophytoplankton, while mesozooplankton were primarily dominated by *Temora* and *Acartia* which could exert equally grazing activity on prey. At this special season, we found that the ciliate clearance rate on phytoplankton was extremely low (Table 1), which dampened the effect of trophic cascades from mesozooplankton. In other words, no matter how the abundance of ciliates was changed by mesozooplankton, the grazing impacts by ciliates were so low as to cause a weak trophic cascade. In the winter, regardless of the composition of mesozooplankton and the abundance of ciliates, both the clearance rates for ciliates and for phytoplankton were rather low, causing extremely low trophic cascades, which were primarily restricted by the low temperature.

To further figure out the driving factors or controlling factors for the change of trophic cascades, we conducted several GAM model analyses with some key factors and the season. The selected significant parameters retained by the final GAM models included environmental factors (temperature and salinity), biological factors (Chl a, ciliates and mesozooplankton dry-weight) and process factors (mesozooplankton clearance rate on ciliates, mesozooplankton ingestion rate on ciliates, and ciliate clearance rate on phytoplankton), respectively, as shown in Table 2. “Deviation explained” is an important indicator for evaluating the performance and validity of the GAM model, helping us to judge the fitting degree and explanatory ability of the model to the data. In this study, the high “deviation explained” (91.5~98%) and *R*^2^ indicate that the parameters we used have well explained the variations of the trophic cascade. At the same time, we observe that the F-values for temperature, ciliate abundance, mesozooplankton clearance rate, and ingestion rate for ciliates, as well as ciliate clearance rate for phytoplankton were quite high. This suggests that they had a strong correlation and a high contribution to the explanation of the models. The spring was a specific season, during which many of the important parameters mentioned above were generally high, thus influencing the change of trophic cascades. The composition of mesozooplankton species was important in explaining the feeding selectivity of the assemblage, which indirectly influenced the trophic cascades discussed above, but they were not computed as factors in analyzing the change of trophic cascades as the data of some important dominant species were not consistently distributed, causing insufficient freedom.

The GAM analysis results of this study indicate that an increase in temperature can lead to an increase in the trophic cascade of mesozooplankton on total phytoplankton and picophytoplankton (Figure 7A). The influence of temperature on the change of trophic cascades on total phytoplankton was basically linearly and positively correlated. This may include the influences on microphytoplankton and nanophytoplankton, although their significance for the models was not recorded. However, the influence on trophic cascades of picophytoplankton was in an inverted U-shaped pattern, indicating that both the low temperature in winter and the high temperature in summer somewhat restricted the predation of zooplankton on the intermediate trophic levels to a certain extent.

The effect of salinity on the change of trophic cascades was not very remarkable, but it significantly influenced the change of trophic cascades on nanophytoplankton. The true reason of why salinity promoted this change in the trophic cascades still needs further research. We think that relatively high salinity in coastal water probably created comfortable environmental conditions for multiple dominant species during the autumn of 2015. Temperature and salinity, two environmental factors, both have corresponding thresholds for adaptation to survival for both meso- and micro-zooplankton, which can also affect their feeding behavior.

The abundance of ciliates significantly influenced the trophic cascade of total phytoplankton, micro- and pico-phytoplankton (Figure 7B). The abundance of ciliates is a key parameter. Firstly, trophic cascades occur as mesozooplankton alters the abundance of ciliates through feeding, thereby influencing the cascading effect. Secondly, mesozooplankton has a distinct preference for ciliates. The higher the abundance of ciliates, the more vigorously mesozooplankton feeds on them, thereby promoting a higher cascading effect. Overall, Chl *a* concentration and mesozooplankton dry-weight were generally not determining factors for the change of trophic cascades of the total phytoplankton community, while they played a notable role in changes in the trophic cascades on nanophytoplankton. The increase of the former will reduce the strength of trophic cascades, while that of the latter will enhance it. We found that a high trophic cascade on nanophytoplankton occurred during the spring of 2017, and meanwhile the concentration of nanophytoplankton was so low that they would inevitably be promoted when their restriction by ciliates was released by trophic cascades. This probably was due to the functional response of ciliates to prey concentrations that the high or low density of phytoplankton will result in a decrease or increase in clearance rate for ciliates and in turn cause a change of trophic cascade. On the other hand, the abundance of ciliates was generally suppressed by the increasing mesozooplankton biomass, thus the trophic cascade will increase when high predation occurs.

GAM analysis showed that feeding rates of mesozooplankton on ciliates and the clearance rate of ciliates on phytoplankton became the most remarkable factors in controlling the strength of trophic cascades, by which the seasonal variation of trophic cascades can be well explained. This finding fulfills the assumptions of this article regarding trophic cascades, and its regression relationship can be directly used for predicting the degree of trophic cascades in the future.

### 4.3. The Significance of Trophic Cascades on Phytoplankton

Due to the presence of microzooplankton, the net feeding of mesozooplankton on phytoplankton can be understood as the combined effect of direct feeding by mesozooplankton and indirect trophic cascades of mesozooplankton on phytoplankton [13,15]. Since the trophic cascades and the direct grazing rates were estimated by this study, now we can evaluate the net role of mesozooplankton in regulating the prey community, including the net growth rate of phytoplankton and the reduction of ciliate grazing impacts on phytoplankton. We did not include heterotrophic dinoflagellates in this study, which may lead to an underestimation of trophic cascades (discussed above). However, if they were included, the total abundance of microzooplankton would increase, meaning the clearance rate of microzooplankton would decrease. And at the same time, because the abundance increases and the $g_{micro}$ we calculated is based on the corresponding abundance, after mutual cancellation, $g_{micro}$ should remain unchanged. That is, the calculation result of the trophic cascade would not be greatly affected. Of course, it would be better if they were included in the calculation, as this might perhaps explain the trophic cascade on diatoms.

Since trophic cascades are in the opposite direction of the feeding rate and in the same direction as the growth rate of phytoplankton, its primary role is to neutralize the amount reduced due to the feeding of mesozooplankton. This is the case in most of this research, such as in the autumn, winter, and the summer of 2015 (Figure 6A). Sometimes it was even higher than the feeding rate (grazing mortality in the figure), such as in the spring of 2017 and the summer of 2016, resulting in an increase of phytoplankton growth. Of course, there are also times when it was less than the feeding rate, mainly because the herbivorous species have an absolute advantage in the composition of mesozooplankton assemblage, resulting in the suppression of phytoplankton growth. This role was acted by the whole mesozooplankton community together, which depends on the proportion of different species with different feeding behavior. It is also controlled by one or two key species or dominant species. For example, Chen et al. (2013) tested several mesozooplankton species in a laboratory showing that the species *P. crassirostris* with a high degree of carnivory caused an increase of phytoplankton, while the cladoceran species *P. avirostris* generally caused a decline of phytoplankton density, and the omnivorous species *T. turbinata* balanced the direct grazing compensation and the indirect trophic cascade effect [15].

We found that whether it is the direct feeding or indirect promotion by mesozooplankton, it is actually insignificant compared to the growth rate of phytoplankton. The grazing impact of mesozooplankton on phytoplankton standing stock is usually less than 10% [3,42], and the average grazing impact calculated in this study was less than 2%. That is to say, trophic cascades have at most increased the growth rate of phytoplankton by 2%. However, the role played by trophic cascades is far more than that. If there is no predation by mesozooplankton, the grazing impacts of microzooplankton could reach 98% without being controlled. But mesozooplankton had reduced it to 70.7% (Figure 8A). This ciliate grazing impact is relatively close to most reports because the results of field investigations were already the consequences after mesozooplankton have exerted trophic cascades [6].

No significant difference of trophic cascades among the three size fractions was found (one-way ANOVA, *p* > 0.05) as there was no significant feeding selectivity for ciliates being observed (Table 1). However, we can see that the difference between trophic cascade and grazing mortality was significantly different among different sizes of phytoplankton. Therefore, the third important role of trophic cascades is to change the structure of prey community. This can be seen from Figure 6C. Except for spring, in many cases, mesozooplankton need to consume a large amount large-sized phytoplankton due to low concentration and resulting in a top-down control to microphytoplankton because of high feeding rate and low trophic cascades. In the spring, because there was already a high concentration of microphytoplankton in the water; mesozooplankton did not need to actively eat them, resulting in strong trophic cascades and leading to the continuation of the spring bloom, instead of this bloom being controlled. At the same time, herbivorous mesozooplankton such as *P. avirostris* were controlling the small-sized phytoplankton in this season. Conversely, in other seasons, when there were fewer large-sized phytoplankton in the water, mesozooplankton exerted a high direct feeding activity, thereby making microphytoplankton occupy only a small proportion of the total phytoplankton in these seasons. And those small-sized particles that cannot be directly consumed by most omnivorous zooplankton were further increased through trophic cascades, allowing them to gain an advantage in seasons with insufficient nutrients or low abundance of the intermediate grazers.

In this study, an in-depth analysis was conducted on the feeding, community structure, and trophic cascades of mesozooplankton in Daya Bay. Daya Bay, as an important marine ecosystem in the northern South China Sea, has unique ecological and limnological characteristics [21]. From an ecological perspective, the water temperature in Daya Bay is relatively suitable, providing a good living environment for many marine organisms. The waters are rich in nutrients, promoting the massive reproduction of plankton and thus providing sufficient food sources for marine organisms at all levels. At the same time, the marine ecosystem of Daya Bay has a certain degree of stability and self-regulation ability, and complex food webs and ecological relationships are formed among different biological groups [22,23,24,25,26]. The seawater salinity in Daya Bay is relatively stable, which is conducive to marine organisms adapting to their living environment. In addition, the hydrodynamic conditions in Daya Bay are complex and are affected by multiple factors such as tides and ocean currents [21]. This complex hydrodynamic environment has an important impact on the distribution and migration of plankton [22]. For example, tidal action and coastal currents can promote the circulation and diffusion of nutrients, thus affecting the growth and reproduction of plankton. It also brings in species like *Canalus sinicus* from the East China Sea [22,26]. Studies have shown that, the structure and function of the planktonic ecosystem in Daya Bay are jointly affected by human activities (such as thermal plant construction and warm water discharge) and natural factors [26]. Mesozooplankton plays a key role in the Daya Bay ecosystem, and its feeding behavior affects the community structure and biomass of phytoplankton through trophic cascades and grazing mortality. At the same time, changes in the community structure of mesozooplankton also reflect the influence of environmental factors. Through the study of trophic cascades, we can better understand the interactions between different biological groups in the Daya Bay ecosystem.

## 5. Conclusions

This study has unveiled several crucial findings pertaining to the role of mesozooplankton in the coastal water food web of Daya Bay. It was revealed that the seasonal variations in the feeding rates and trophic cascades of mesozooplankton are influenced by multiple factors. The clearance rate of mesozooplankton is impacted by various elements, including the biomass of phytoplankton, mesozooplankton feeding preferences, and composition. For instance, the composition of mesozooplankton species in different seasons significantly contributes to these variations. Furthermore, the abundance of ciliates and environmental factors such as temperature and salinity play pivotal roles in determining the intensity of trophic cascades. We also further underscore the significance of trophic cascades in regulating the prey community by balancing the direct grazing mortality and trophic cascades, by altering the composition of different sizes of phytoplankton, and by reducing the grazing impacts from ciliates on phytoplankton. However, further research is needed to elucidate the detailed relationship between the feeding habits of mesozooplankton and trophic cascades. In summary, this research offers valuable insights into the marine food web, but additional studies are required to enhance our understanding and inform the management and conservation of marine ecosystems in the context of environmental changes.

## Figures and Tables

**Table 1:** Grazing rate and clearance rate of ciliates on different sizes of phytoplankton.

Year	Season	$\mathrm{Grazing}\;\mathrm{Rate}\;(g_{micro}$) (d^−1^)				Clearance Rate (*f_micro_*) (mL·ind.·^−1^·d^−1^)			
		>20 μm	2–20 μm	<2 μm	Total Chl	>20 μm	2–20 μm	<2 μm	Total Chl
2015	Spring	1.82	1.14	1.76	1.63	0.85	0.53	0.82	0.77
	Summer	0.85	0.96	0.68	0.83	0.78	0.88	0.63	0.77
	Autumn	0.63	0.27	0.50	0.45	0.20	0.09	0.16	0.14
2016	Winter	0.10	0.75	0.16	0.36	0.41	3.35	0.69	1.61
	Summer	1.00	1.11	1.03	1.17	1.64	1.81	1.68	1.92
	Autumn	0.26	0.68	1.68	0.22	0.14	0.38	0.93	0.12
2017	Winter	1.08	1.82	1.21	1.71	0.46	0.78	0.52	0.73
	Spring	2.27	1.64	1.55	2.06	2.83	2.04	1.93	2.57

**Table 2:** GAM analysis for mesozooplankton trophic cascades on phytoplankton.

Predictors	Total Phytoplankton		Chl *a* > 20 μm		Chl *a* 2–20 μm		Chl *a* < 2 μm	
	F/t	Sig.	F/t	Sig.	F/t	Sig.	F/t	Sig.
Spring	7.345	<0.001 **	5.416	<0.001 **				
Summer					−6.838	<0.001 **		
Autumn								
Winter					−4.200	<0.001 **		
Temperature	14.950	<0.001 **	5.732	ns	0.181	ns	12.467	<0.001 **
Salinity	2.237	ns	0.284	ns	32.888	<0.001 **	0.105	ns
Chl *a*	2.721	ns	0.145	ns	14.546	0.005 **	0.488	ns
Ciliates	20.769	<0.001 **	8.033	0.018 *	0.623	ns	8.312	0.012 *
Mesozooplankton dry-weight	0.025	ns	3.608	ns	5.655	0.043 *	0.216	ns
Mesozooplankton clearance rate on ciliates	20.447	<0.001 **	3.172	ns	29.676	<0.001 **	6.013	0.008 *
Mesozooplankton ingestion rate on ciliates	6.364	0.009 **	11.791	0.001 **	19.694	<0.001 **	10.182	0.007 **
Ciliate clearance rate on phytoplankton	17.699	<0.001 **	17.557	0.001 **	23.303	0.001 **	2.005	ns
Deviance explained (%)	97.8%		97.8%		98%		91.5%	
R^2^	0.963		0.962		0.965		0.854	

Note: ** indicates significance level at <0.01; * indicates significance level at <0.05; “ns” indicates no significant correlation.

## Data Availability

The original contributions presented in the study are included in the article, further inquiries can be directed to the corresponding authors.
